# Challenges in diagnosing and managing Ewing sarcoma in older adults: a case report of Ewing sarcoma presenting as seemingly uncomplicated back pain

**DOI:** 10.1093/jscr/rjaf489

**Published:** 2025-07-11

**Authors:** Haley N Bayne, Helen Ellsworth, Esma Karlovich, Osama Al Dalahmah, Vadim Tikhomirov

**Affiliations:** Department of Medicine, Robert Larner College of Medicine at the University of Vermont, 89 Beaumont Ave, Burlington, VT 05405, United States; Department of Pathology at Columbia University Medical Center, 622 W 168th St, New York, NY 10032, United States; Department of Pathology at Columbia University Medical Center, 622 W 168th St, New York, NY 10032, United States; Department of Pathology at Columbia University Medical Center, 622 W 168th St, New York, NY 10032, United States; Department of Internal Medicine at Danbury Hospital, 24 Hospital Ave, Danbury, CT 06810, United States

**Keywords:** Ewing sarcoma, older adults, intradural tumor

## Abstract

Ewing Sarcoma (ES) is a rare, aggressive malignancy typically seen in younger people, with diagnosis in older adults being uncommon. We present a case of a 70-year-old woman with ES. She presented with a common complaint of referred lower extremity pain that started without injury. However, unusual clinical features included generalized pruritus and nocturnal pain. Thus, it was decided to proceed with imaging. MRI revealed an intradural L3–L4 mass, initially suspected to be a schwannoma, considering homogenous contrast enhancement and no sign of invasion of the surrounding tissues. Her condition was not improving and was unresponsive to oral steroids. Surgical intervention yielded a pathology report confirming ES. She underwent radiation and chemotherapy but experienced complications, including thrombocytopenia and neutropenia. ES in older adults is rare, often presenting atypically and carrying a guarded prognosis. This case underscores the need for age-specific protocols and improved management strategies.

## Introduction

Ewing sarcoma (ES) is a rare, aggressive cancer that peaks between ages 10 and 20. It features small, round, blue cells, and a characteristic t(11;22) translocation producing the Ewing sarcoma breakpoint region 1/Friend leukemia integration 1 transcription factor (EWS/FLI1) fusion gene, a driver of tumorigenesis [1]. No clear links have been found between ES and environmental, medical, or familial risk factors [2], though the T309G polymorphism of O6-methylguanine-DNA methyltransferase (MGM2)—common in Ashkenazi Jewish populations—may confer susceptibility [3, 4].

Multimodal therapy has improved pediatric survival for localized ES to 70%–80% [5], but adult outcomes, especially over age 40, are worse and understudied. ES typically affects bones (long bones, pelvis) but 25% are extraosseous, more common in adults, and often arise in the chest wall, muscles, or connective tissue [6]. Radiographically, ES shows aggressive bone destruction, “onion-skin” periosteal reaction, and soft tissue invasion—unlike benign tumors such as lipomas and schwannomas [7].

Adults over 40 account for <1% of cases and have worse prognoses due to delayed diagnoses and higher metastatic rates, with some studies citing a 5-year survival of just 37% [8, 9], compared to 70% in children [1]. Symptoms include localized, often nocturnal, pain; systemic signs like fever and weight loss suggest metastasis [2].

Treatment depends on disease extent. Surgical resection is preferred for localized tumors, while chemotherapy remains essential, though less well tolerated in older adults due to toxicity [10].

## Materials and methods

This case report describes a 70-year-old female who presented with a history of progressive right leg pain and generalized pruritus, ultimately found to have imaging evidence of an intradural spinal tumor. She underwent surgery for subtotal tumor resection, and subsequent histopathological analysis confirmed the diagnosis of ES. A retrospective review of her clinical presentation, diagnostic workup, and clinical course was performed. Outcomes were assessed based on symptom evolution, post-operative recovery, and final pathology. Additionally, a literature review was conducted to contextualize the rarity of intradural ES in older adults.

## Results

A 70-year-old female with a history of polymyalgia rheumatica, osteopenia, hypercholesterolemia, hypertension, gastroesophageal reflux disease, and atypical epithelial hyperplasia of the right breast presented with progressive right leg pain and generalized pruritus. Initially intermittent, the pain became severe, disrupting sleep.

**Figure 1:**
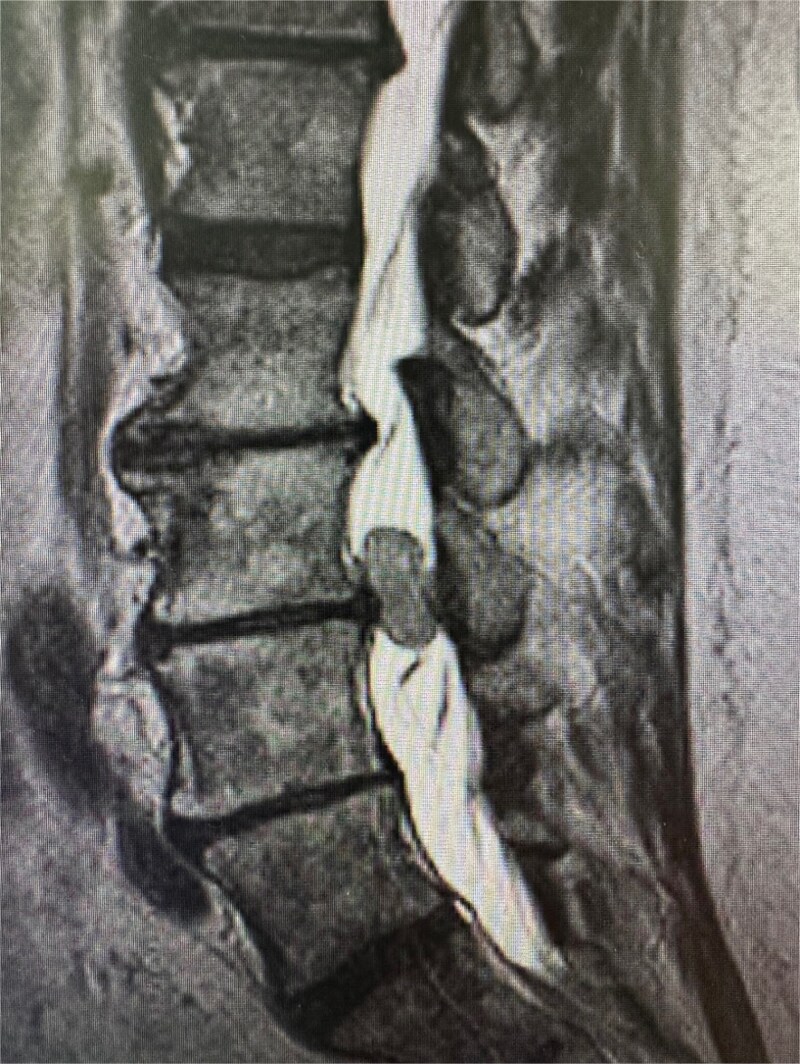
Sagittal T2-weighted MRI of the lumbar spine demonstrating a well-circumscribed intradural extramedullary mass measuring 0.9 × 1.4 × 1.9 cm at the L3–L4 level. The lesion exhibits intermediate signal intensity.

Physical examination was unremarkable, except for tenderness at the right sacroiliac joint. Neurological testing revealed normal reflexes and strength. Sagittal T2-weighted MRI of the lumbar spine demonstrating a well-circumscribed intradural extramedullary mass measuring 0.9 × 1.4 × 1.9 cm at the L3–L4 level. The lesion exhibited intermediate signal intensity (Fig. 1). Other differential diagnoses at this time included meningioma, cystic lesions, infectious processes, or metastases. The patient was referred to neurosurgery.

The patient underwent a subtotal tumor excision via laminectomy. The tumor was closely adherent to the cauda equina nerve roots, precluding complete resection.

**Figure 2:**
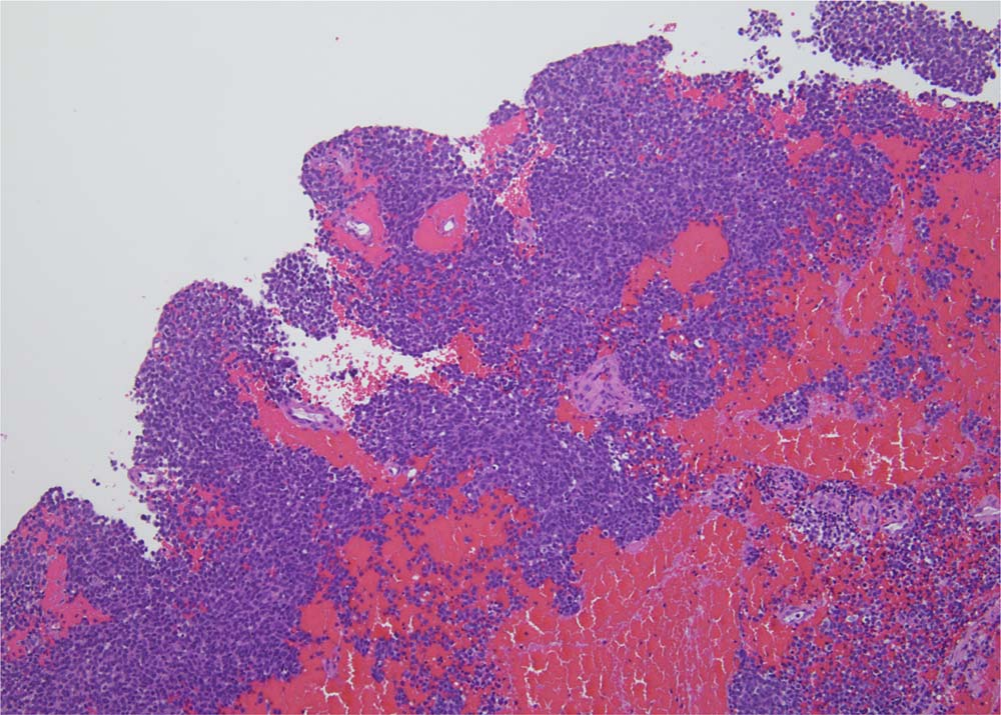
H&E, 10×. The biopsy shows sheets of small round blue cells admixed with blood and fibrous tissue.

**Figure 3:**
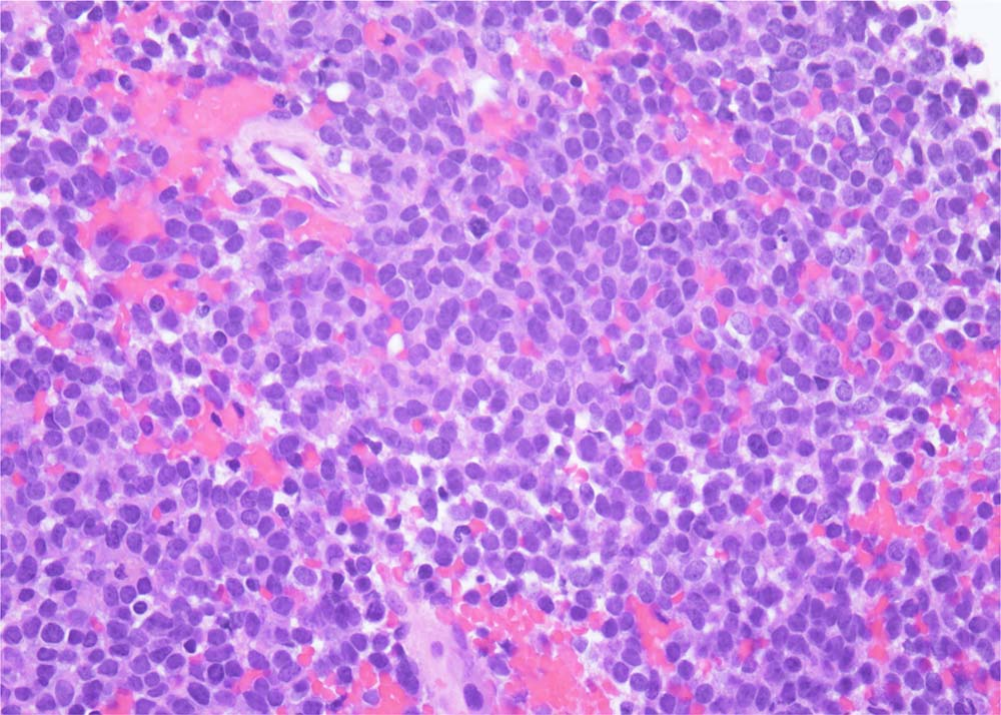
H&E, 40×. The neoplastic cells have scant cytoplasm, finely stippled chromatin, and inconspicuous nucleoli. At high power, mitotic figures and apoptotic debris are visible.

Histopathology revealed small, round, blue cells (Fig. 2), and other findings consistent with ES (Fig. 3).

**Figure 4:**
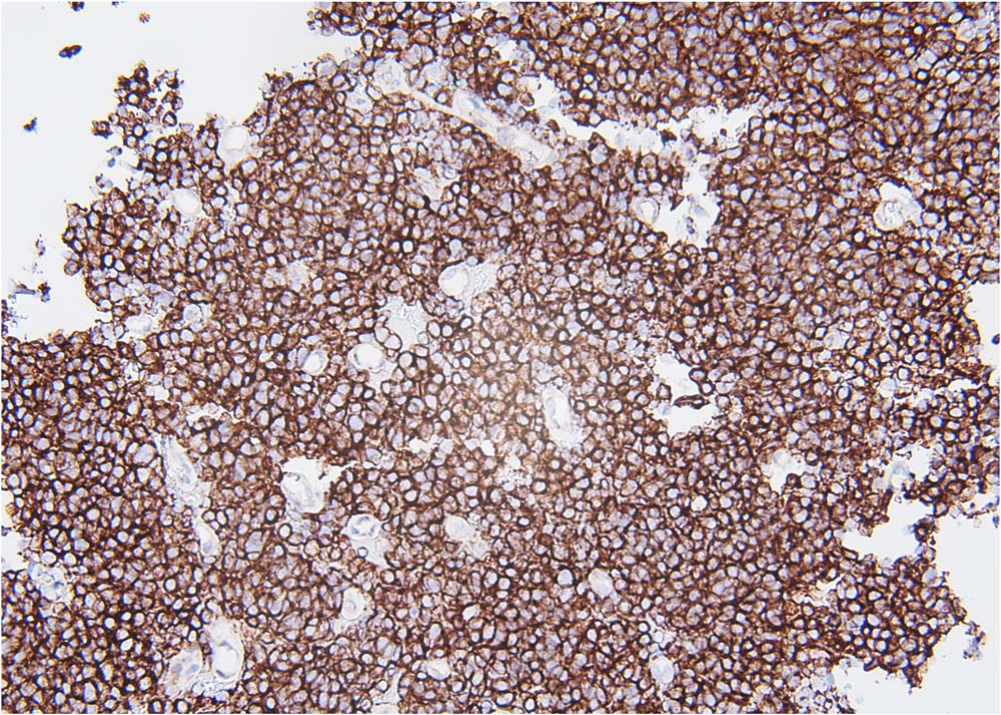
CD99, 20×. Immunohistochemical stain with CD99 demonstrates diffusely positive membranous staining of neoplastic cells.

Molecular staining further supported an ES diagnosis (Fig. 4). Immunohistochemical staining with NKX2.2, which is highly specific for ES, demonstrated positive nuclear staining of neoplastic cells. Furthermore, molecular testing confirmed the presence of an EWS fusion.

Postoperatively, the patient retained normal neurological function but reported persistent leg pain. The patient initiated chemoradiation following the Ewing’s protocol. She received alternating vincristine-cyclophosphamide and ifosfamide-etoposide every 3 weeks. Following the second chemotherapy cycle, she was hospitalized for thrombocytopenia requiring platelet transfusion and neutropenia. She continued radiation therapy concurrently.

## Discussion

### Diagnostic considerations of back pain and ES in older adults

This case exemplifies a very common presentation—back and leg pain—with the very uncommon diagnosis of intradural ES. This case illustrates that persistent radicular symptoms, despite a short course of steroids, as well as other systemic systems, such as pruritus, should prompt reconsideration. Clinicians must remain mindful that epidural or intradural masses—though rare—can underlie persistent symptoms.

ES in adults, particularly those over 40, presents unique challenges due to delayed diagnosis, comorbidities, and decreased treatment tolerance. Age is a key prognostic factor, with survival rates significantly lower than in pediatric cases [1]. Current ES treatment guidelines remain largely pediatric-derived, despite older patients’ increased vulnerabilities [8].

In contrast to existing studies on adult ES that highlight several factors predicting poor prognosis, such as metastatic disease at presentation, extraosseous primary tumors, and larger tumor size [1, 9], our patient did not exhibit any of these indicators. She presented with localized disease, and no metastasis was detected at the time of diagnosis. Additionally, her tumor was not extraosseous, and it did not involve excessively large dimensions. Furthermore, her serum markers, including lactate dehydrogenase and alkaline phosphatase, were not notably elevated, suggesting a less aggressive course compared to those often associated with poorer outcomes in adults with ES.

### Atypical features and prognostic implications

The generalized pruritus seen in our patient in her disease presentation may suggest underlying systemic or paraneoplastic processes caused by her spinal ES, which has not been previously described in the literature. Although uncommon, pruritus can occur as part of a paraneoplastic syndrome due to cytokine release or immune dysregulation triggered by the tumor [11]. Additionally, systemic inflammation driven by elevated cytokines from the tumor microenvironment remains a potential contributor, even in the absence of abnormal laboratory findings. These differences underscore the variability in clinical presentations and outcomes of ES, even within the adult population.

### Review of paraneoplastic syndromes

More broadly, paraneoplastic processes are rare disorders triggered by an immune response to an underlying malignancy, leading to systemic effects not directly caused by the tumor itself. Symptoms can affect multiple organ systems, inducing neurological, endocrine, dermatological, and hematological symptoms [12]. Recognizing these syndromes is crucial, as they can manifest before or after a cancer diagnosis and may serve as early indicators of hidden malignancies. Early detection and management of these syndromes can be challenging, especially in patients who exhibit limited systemic signs, like the patient in this case report.

## Conclusion

This case underscores the diagnostic complexity of ES in older adults, particularly when the presentation mimics benign spinal pathology. The intradural location and relatively indolent imaging features initially obscured the diagnosis. Additionally, the patient’s unexplained pruritus may represent a paraneoplastic manifestation, which is rare and previously undescribed in ES. This highlights the importance of maintaining a broad differential diagnosis—even when imaging appears non-aggressive—and recognizing that subtle systemic symptoms, such as associated pruritis, may point to an underlying malignancy. Clinicians should remain alert to atypical presentations, particularly in older patients, to ensure timely diagnosis and appropriate management.
